# Will the central land inspection affect the intensity of local environmental protection? Evidence from China

**DOI:** 10.1007/s11356-023-31672-5

**Published:** 2024-01-15

**Authors:** Shichang Ma, Yuan Zhang

**Affiliations:** School of Urban Economics and Management, Beijing University of Civil Engineering and Architecture, Beijing, 100044 China

**Keywords:** Land inspection, Environmental pollution, Difference-in-differences model, Local fiscal revenue

## Abstract

Observing the impact and alienation of local government implementation of central policies is helpful for a comprehensive understanding of the effectiveness of central policy execution and the systematic formulation of central-local management policies. This paper takes the influence of land inspections on local government environmental behavior as the research object, based on the land inspection system initiated by the central government in 2006. It uses panel data from 30 provinces in China and a difference-in-differences method to assess the environmental protection crowding-out effect of land inspections for the first time and conducts an in-depth exploration of its mechanisms and heterogeneity. The study findings are as follows: (1) In a long-term sample spanning from 1997 to 2020, the establishment of land inspection bureaus did not significantly increase the level of environmental pollution in the host regions. (2) In a short-term sample spanning from 2000 to 2015, the establishment of land inspection bureaus significantly increased the level of environmental pollution in the host regions. (3) The environmental protection crowding-out effect of land inspections is mainly due to the restraint on local government fiscal revenue. (4) The larger the economic scale, the more significant the positive effect of land inspections on the level of environmental pollution in the host regions.

## Introduction

Land and the environment are core resources for local economic development, and they also pose significant constraints on local economic growth. The coordination between land resources and environmental protection is a crucial decision-making challenge for local governments. Since the reforms in the tax-sharing system and the marketization of housing, local governments have gradually relaxed their control over land and environmental resources in response to inter-regional competition and other pressures, while vigorously promoting local GDP growth (Chen et al. 2015; Li 2014). In contrast, the central government has introduced stringent regulatory measures to address these issues.

In the field of land management, as illegal land activities have been on the rise, there was a need to improve China’s land law enforcement and oversight system (Lian et al. 2019; Yin et al. 2020). In July 2006, the State Council issued a notice titled “Regarding the Establishment of the National Land Inspection System,” officially establishing the land inspection system in China (State Council, People’s Republic of China 2006). The institutions responsible for land inspection include the National Land Inspectorate Office and nine dispatched National Land Inspection Bureaus in various regions, namely, Beijing, Shanghai, Nanjing, Jinan, Wuhan, Guangzhou, Chengdu, Xi’an, and Shenyang. These National Land Inspection Bureaus are tasked with supervising and inspecting land law enforcement on behalf of the National Land Inspectorate Office (Zuo 2007). The study of the establishment of National Land Inspection Bureaus and their impact on local environments, as well as their role in mitigating and addressing adverse environmental effects, holds significant theoretical and practical significance.

In this paper, we attempt to investigate whether the establishment of National Land Inspectorate will increase the level of environmental pollution in the resident area. The establishment of the land inspection system is intended to strengthen the central government’s supervision and control over local land resource management. However, some scholars argue that although local governments in China actively promote economic development, they have failed to avoid short-sightedness, which has had an impact on long-term growth. One reason for this is the inherent monitoring problem between the unitary government and the central-local division of labor, characterized by principal-agent relationships (Naughton 2016; Zhou 2008). Additionally, the Chinese government system is designed to incentivize officials, leading to excessive pursuit of political achievements. Land resource management serves as a tool for short-term fiscal revenue control and offers optional strategic flexibility for local governments (Geng et al. 2018). Land resources play a significant role in the fiscal economy of local governments through a combination of “land finance” and “investment attraction” activities (Zhu et al. 2019). In the context of the decentralization of powers between the central and local governments and the competitive pressures faced by local governments, the mismatch between fiscal powers and administrative powers has led to a “structural” fiscal power gap, and the competition between governments has generated a “competitive” fiscal power gap (Tang et al. 2019). These gaps place significant fiscal pressures and competitive challenges on local governments. When faced with negative shocks that simultaneously affect economic growth and fiscal revenue, such as land inspections, local governments become more cautious in adjusting the intensity and methods of policy implementation. They tend to allocate fiscal resources to expenditure projects that can yield visible political achievements. This can result in a lack of coordination between local government policies related to central land inspection and local environmental protection (Liu and Peng 2022), creating a “rob Peter to pay Paul” policy crowding-out effect. Consequently, this can impact the effectiveness of central policies and run counter to the goal of high-quality development (Zhong and Zheng 2022). As a result, research is conducted to explore whether the establishment of land inspection bureaus exacerbates the level of local environmental pollution.

Currently, there is a substantial body of literature on the factors influencing environmental pollution, with many studies evaluating and analyzing the effects of these factors. Sun et al. (2017a) argue that population growth, economic expansion, and urbanization will exacerbate provincial-level environmental pollution, while upgrading industrial structures and increasing openness to the outside world will help alleviate provincial-level environmental pollution. Yuan et al. (2019) found that there is a clear spatial clustering pattern in environmental quality, highlighting regional imbalances, and the trend of environmental deterioration has not been fundamentally reversed. In the eastern regions, there is a gradual coordination between environmental quality and economic development, but population concentration has a significant negative impact on the environment. In the central and western regions, the “high investment, high energy consumption, high pollution, low efficiency” development model is a constraint on improving environmental quality. Sun and Gao (2021) found a significant “inverted N-shaped” relationship between economic concentration and environmental pollution and a relatively weak “inverted U-shaped” relationship between energy intensity and environmental pollution. However, there is currently a lack of research on the impact of the establishment of land inspection bureaus on the local environment. In traditional policy practice, whether it is environmental policy or land policy, research and policy formulation are mainly conducted as a single entity in a relatively closed environment. This paper aims to comprehensively and systematically improve environmental protection and land policies from the perspective of local government policy implementation. It constructs a long-term mechanism for coordinating ecological civilization and economic development, including top-level design, regulatory policy systems, macroeconomic control measures, environmental regulation, land behavior control, evaluation and assessment, etc. This paper fills the gap in current research by integrating environmental protection and land resource management policies into a unified analytical framework and studying dynamic policy combinations rather than single policies.

A scientific assessment of the impact of land inspection bureau establishment on environmental pollution is an important aspect of understanding the effectiveness of the land inspection system. This research can contribute to further refining the relationship between the land inspection system and environmental protection. Taking the establishment of the land supervision bureau as a quasi-natural experiment, this paper empirically investigates the environmental protection crowding-out effect of land supervision using panel data from 30 provinces in China from 2000 to 2015 through the difference-in-differences model. It further explores the mechanisms and heterogeneity of this effect. The contribution of this paper lies in studying the coordination issue between central policy formulation and local government implementation from the perspective of principal-agent relationship and local government incentives. The research results will help to comprehensively understand the behavioral motivations of local governments and systematically adjust the incentive and constraint system between the central and local governments, thereby promoting high-quality macroeconomic development based on the micro-actions of local governments.

## Theoretical hypothesis

Looking back at the supply background of the national land supervision system, it is not difficult to find that the central government has given important resource allocation and administrative supervision powers to the land supervision agencies stationed in local areas.

Firstly, the land supervision agencies have the authorized power and authority from the central government to conduct supervision of land management in their respective areas through various policy tools and measures. The “Notice of the State Council of the People’s Republic of China on the establishment of the national land supervision system” clearly stipulates that local governments should actively support and cooperate with the national land supervision agencies in their work. From the perspective of resource acquisition and political promotion, local governments have strong political incentives to cooperate with the central government in land supervision (Aidt and Dutta 2004; Zhang et al. 2012; Zhang and Zhou 2008). This is because there is information asymmetry when the supervisory agency carries out its duties, and the active cooperation of local governments may serve as a signal showing their good political reputation (Zheng and Zhu 2013), which helps local governments increase their political capital.

Secondly, the power deterrence of the land supervision agencies stationed in local areas may be stronger than in other areas within their jurisdiction. Geographic distance is an important indicator of measuring the degree of information asymmetry (Huang et al. 2017); for supervisors, the closer they are to the supervisees, the more obvious their advantage in obtaining information. On the contrary, the farther the distance is, the higher the communication cost, and the more difficult it is for supervisors to obtain information from the supervisees (Bloom et al. 2014). Therefore, the advantage in information resulting from proximity is an important factor in the political deterrence of government institutions. In this paper, because the land supervision bureau assigned by the central government to local areas has administrative supervision powers, the administrative center (stationed location) may become an important geographical factor affecting the degree of land violations. The areas closer to the location of the supervision bureau may have a stronger advantage in acquiring information about land violations, thus leading to greater regulatory deterrence and a decrease in land violations. On the contrary, the areas farther away from the location of the bureau may face higher communication costs, resulting in weaker regulatory deterrence and a relatively more serious situation of land violations. In other words, the resident effect of the land inspector bureau is very obvious.

More importantly, in recent years, with the rapid development of China’s economy and society, the contradiction between land supply and demand has become increasingly prominent, and various types of land violations have remained high. These violations exhibit a prominent feature: they are mostly led by local governments. Since the revision of the Land Administration Law in 1998, violations directly involving local governments have consistently accounted for over 30% of the total, and almost all serious land violations are related to local governments; moreover, 50% of violations by enterprises and institutions would be difficult to implement without the tolerance and cooperation of local governments (Liang 2010). Therefore, to effectively curb and address land violations, it is crucial to fully understand the land utilization behavior of local governments and their motivations for violations. Previous studies have shown that regional economic growth (Long and Chen 2011), land market reforms (Chen and Wang 2013), and changes in central government regulatory policies all have an impact on the scale of local land violations (Li et al. 2013). However, the financial incentives formed by the large amount of land transfer fees (Long 2013) are the main motivation for local government land violations. Specifically, since the fiscal decentralization reform, the “fiscal powers go up while administrative powers stay at the lower level” has resulted in significant fiscal deficits for local governments. In this situation, land finance has become an important source for local governments to expand their fiscal resources, and it is referred to as the “second budget” (Sun and Zhou 2013). The pathways through which land resources impact fiscal conditions have two aspects: Kostka (2013) suggests that land transfer fees constitute a significant source of local fiscal revenue after the tax-sharing reform, emphasizing the importance of “land finance” in local finances. Yu et al. (2016) highlights that local governments attract more businesses by offering land at lower prices, emphasizing the “investment attraction” aspect. Gai (2012) argues that the existing fiscal system is a factor that incentivizes illegal land activities in the public sector. Deng and Zhang (2019) found that increasing the degree of marketization in land resource allocation can effectively curb illegal land activities. Huang et al. (2020) empirically studied the interactive relationships between the land market, fiscal pressures, and economic growth. Due to the fact that local governments are the actual controllers of land resources within their jurisdictions and can easily intervene in the processes of land acquisition, reserves, and allocation through administrative means (Guo and Wang 2013), land finance essentially involves collecting future land rents for a certain number of years. Local governments often engage in short-sighted behavior of selling land quickly and excessively, leading to the expansion and occupation of urban land becoming the actual mechanism for increasing local fiscal revenue (Lou and Wang 2013). When this mechanism conflicts with strict national land control policies, local governments, driven by fiscal incentives, inevitably engage in illegal land activities. When local governments implement central land inspection policies, they may allocate fiscal resources to projects that can yield visible political achievements. This could lead to a relative reduction in local investments in environmental pollution control measures, potentially weakening efforts in local environmental governance.

The effectiveness of central land inspection implies that it compels local governments to supply land in compliance, reduce illegal encroachments on arable land, decrease agreements for land transfer, and improve land procedures (Wang et al. 2021). However, in the short term, it can significantly reduce the total fiscal revenue of local governments. Under the pressures of “structural” fiscal power gaps and “competitive” fiscal power gaps (Xu 2019), and in an information environment characterized by information asymmetry between central and local authorities, local governments may relax environmental regulations to improve their fiscal and economic conditions in response to central land inspection.

Therefore, it can be concluded that compared to non-residential areas, the land inspection agencies stationed in local areas have greater authority and deterrence power, resulting in lower levels of land violations. Local governments in these stationed areas are more likely to relax environmental regulations to improve their fiscal and economic conditions. Looking back at the supply context of the national land inspection system, it is evident that the central government has given these inspection agencies significant power in resource allocation and administrative supervision in their stationed regions. By enhancing the authority of these inspection agencies, they are better equipped to represent the central government in carrying out inspection duties and rectifying issues related to land management. Based on this, this paper proposes the following research hypotheses:

- *H1: The establishment of land inspection agencies, compared to non-residential areas, will relatively increase the level of environmental pollution in the stationed areas.*
- *H2: The establishment of land inspection agencies affects the level of local fiscal revenue, which in turn influences the level of local environmental pollution.*

This paper divides the sample into eastern and central-western regions based on the provinces’ geographical locations and conducts group regression analysis to empirically test the location heterogeneity in the impact of the establishment of land inspection agencies on the level of environmental pollution in stationed areas. Guo et al. (2018) found significant regional differences in environmental pollution among provinces, primarily due to the uneven economic development levels in eastern, central, and western regions of China. Research by Tang et al. (2022) found that the overall level of regional environmental governance shows a pattern of decreasing from east to west. Hu and Zhang (2020) suggested that environmental regulations in the eastern region are enforced more vigorously and effectively, leading to significant pollution control outcomes. In contrast, environmental regulations in the central and western regions have not achieved significant pollution control results. The eastern region’s high environmental regulation intensity and enforcement effectiveness can lead businesses to relocate to the central-western regions due to cost considerations, resulting in increased pollution in the western region. Western governments, driven by economic performance considerations, may not enforce environmental regulations as rigorously as in the eastern region. Compared to the central-western regions, the stronger environmental regulations and enforcement in the eastern region can result in better environmental governance outcomes in China (Yang and Zheng 2013). Therefore, the establishment of land inspection bureaus may have a different impact on the intensity of environmental regulations in the eastern and western regions. Based on this, this paper proposes the third research hypothesis:

- *H3: There is location heterogeneity in the environmental impact of the establishment of land inspection agencies.*

## Research design

### Variable selection

1. Dependent variable

The comprehensive environmental pollution index is selected as the dependent variable. In this study, the entropy weighting method proposed by Xu and Deng (2012). is used to calculate the comprehensive environmental pollution index. A higher index value indicates more severe environmental pollution. The calculation formula and weights for the entropy weight method can be found in Appendices 1 and 2 The index is computed using four environmental pollution metrics: industrial wastewater discharge, industrial sulfur dioxide emissions, smoke (dust) emissions, and industrial solid waste emissions, with the entropy weighting method.

(2) Core independent variable

The cross-product of the national land inspection bureau stationed in a province (prov) and event year indicator (year). If a province has established a national land inspection bureau, it is assigned a value of 1; otherwise, it is assigned a value of 0. Since there are nine national land inspection bureaus established by the country, this implies that nine provinces are assigned a value of 1, while the rest are assigned a value of 0. The national land inspection system was officially implemented in 2007. If a province is in the event year (2007) or later, we assign a value of year = 1; otherwise, year = 0.

(3) Control variables

- Economic development level: This study measures the level of economic development using per capita real GDP. A higher per capita real GDP indicates a higher level of economic development in the local area.
- Industrial structure: In the early stages of economic development, an increase in the pace of industrialization often leads to excessive resource exploitation and a significant increase in waste emissions. When the economy reaches a certain stage of development, the growth pattern gradually shifts from extensive to intensive, and the industrial structure undergoes corresponding optimization and upgrades. The share of industry in the national economy decreases, and the share of the tertiary sector rapidly increases, resulting in reduced resource and environmental pressures. This study uses the proportion of the value of the secondary sector to the regional gross domestic product (GDP) to measure industrial structure.
- Environmental governance intensity: This study measures the intensity of environmental governance using the amount of investment in industrial pollution control. A higher investment in industrial pollution control indicates a higher level of environmental governance intensity.
- Energy intensity: This study measures energy intensity using energy consumption per unit of GDP.

(4) Mediating variable

This study uses the annual growth rate of local government fiscal revenue (revenue) as the measure of the mediating variable “fiscal revenue level.”

The specific names, symbols and calculation methods for each variable are detailed in Table 1.

**Table 1:** Variables and definitions

Variable type	Variable name	Symbol	Variable description
Dependent variable	Comprehensive environmental pollution index	P	The index is computed using four environmental pollution metrics: industrial wastewater discharge, industrial sulfur dioxide emissions, smoke (dust) emissions, and industrial solid waste emissions, with the entropy weighting method.
Core independent variable	Virtual variable for the location of the National Land Inspection Bureau	prov	It takes the value of 1 if the province has established a land inspection bureau, otherwise 0.
	Event year virtual variable	year	If the province establishes an inspection bureau in the event year or later, it takes the value of 1, otherwise 0.
	Interaction term	DID	prov_*it*_ × year
Control variables	Economic development level	lnECO	Natural logarithm of per capita real GDP.
	Industrial structure	industry	Industrial output value of the secondary industry/regional GDP.
	Environmental governance intensity	lnENV	Logarithm of industrial pollution control investment.
	Energy intensity	energy	Energy consumption per unit of GDP.
Mediating variable	Annual growth rate of local government fiscal revenue	revenue	(Current year’s local government fiscal revenue − previous year’s local government fiscal revenue) / previous year’s local government fiscal revenue × 100%.

### Model specification

Referring to the study by Razzaq et al. (2023), to address the endogeneity issue of variables, the establishment of the land supervision bureau in 2007 is treated as a “quasi-natural experiment.” The main influencing factors are taken as control variables, and a difference-in-differences (DID) model is constructed to compare and analyze the differences in environmental indicators between areas before and after 2007 and at different distances from the land supervision bureau. This aims to identify the impact and causal relationship of the land supervision bureau on environmental pollution and examine the existence of the “environmental pollution effect of land supervision.” Specifically, we consider provinces where the national land supervision bureau is established as the “treatment group” and all other provinces without the establishment of a land supervision bureau as the “control group.” To control for systematic differences between the control and treatment groups, we designate 2007 as the “event year.” If a province belongs to the treatment group, we assign prov = 1; otherwise, prov = 0. Similarly, if a province is in the event year or later, we assign year = 1; otherwise, year = 0. Constructing a panel difference-in-differences estimation equation model as follows:

1 $${y}_{it}={\beta}_0+{\beta}_1\ {\textrm{prov}}_{it}\times \textrm{year}+{\beta}_2{z}_{it}+{\alpha}_i+{\gamma}_t+{\varepsilon}_{it}$$

In the above equation, the subscripts *i* and *t* represent the *i*th province and the *t*th year, respectively. *y*_*it*_ represents the dependent variable, indicating the level of environmental pollution. Borrowing from the research by Xu and Deng (2012), this study uses the “comprehensive environmental pollution index” to measure the level of environmental pollution. *α*_*i*_ represents individual fixed effects for each province, *γ*_*t*_ represents time fixed effects, and *ε*_*it*_ is the random error term. *z*_*it*_ includes other control variables, such as economic development level, industrial structure, environmental governance, and environmental regulatory intensity. The estimate of the cross-term coefficient *β*_1_ of the two dummy variables in model (1), which is mathematically equal to the difference between the experimental group before and after the event year minus the difference between the control group before and after the event year, is the focus of our attention; this is known as “difference-in-differences.” Additionally, this study employs a panel difference-in-differences model to estimate by taking within-group differences, thereby controlling for unobserved effects. If the environmental pollution level within the jurisdiction of the land inspection is indeed higher than in non-resident areas, then the coefficient of *β*_1_ should be significantly positive, indicating that the establishment of the land inspection bureau exacerbates local environmental pollution.

### Data source and description

This study used panel data from 30 provinces across China spanning from 1997 to 2020. Due to data availability, Tibet Autonomous Region, Hong Kong, Macau, and Taiwan Province were not included. The data sources primarily included the “China Statistical Yearbook,” “China Environmental Statistical Yearbook,” “China Environmental Bulletin,” and provincial statistical yearbooks. Additionally, data were obtained from the official websites of the National Bureau of Statistics and the National Environmental Protection Agency. For the small amount of missing data, interpolation method is used for completion. Table 2 reports the descriptive statistics of the main research variables.

**Table 2:** Descriptive statistics of variables

Variable	Mean value	Standard deviation	Minimum value	Maximum value
P	0.0332	0.0053	0.0240	0.0446
DID	0.1687	0.3749	0.0000	1.0000
lnECO	9.8771	0.7804	7.9226	11.6497
lnENV	11.4717	1.1293	6.9137	14.1637
energy	1.3932	0.7981	0.2745	4.5526
industry	0.4429	0.0801	0.1784	0.6567
revenue	0.1896	0.1055	-0.3337	0.5838

## Empirical analysis

### Baseline regression results

Selecting the sample time frame from 1997 to 2020, we conducted baseline regression analysis. We controlled for location and time effects and included control variables in the regression. The regression results are shown in Table 3.

**Table 3:** Baseline regression result

Variable	Regression coefficients	*t*-value	*p*-value
DID	0.0009	1.2200	0.2220
treat	0.0032	5.5400	0.0000
post	0.0022	3.9500	0.0000
lnECO	−0.0016	-5.3200	0.0000
lnENV	0.0001	3.6500	0.0000
energy	−0.0009	-3.7100	0.0000
industry	0.0306	16.4700	0.0000
Constant term	0.0348	11.5800	0.0000
R-squared	0.3195		
Number of obs	720		

The results in Table 1 indicate that the coefficient for the “DID” variable is 0.0009, which is not statistically significant (*p* > 0.1, *t* < 2). This suggests that during the time period from 1997 to 2020, the first research hypothesis was not supported.

The reasons for this result may be as follows: First, the establishment of the land inspection system and the Land Inspection Bureau in 2006 means that over time, the environmental impact may have diminished. Second, in recent years, China has continuously strengthened environmental regulation, with the introduction of policies such as the “Environmental Protection Law,” environmental authorities summoning top local government officials, the “Air Pollution Prevention and Control Law,” and initiatives like the “Ten Measures for Air,” “Ten Measures for Water,” and “Ten Measures for Soil,” all of which have weakened the impact of land resource control.

Based on this, the study further narrows down the sample space by selecting panel data from 30 provinces in China from 2000 to 2015 to construct a difference-in-differences model for empirical testing. We controlled for location and time effects and included control variables in the regression. Some control variables were found to be not statistically significant. We removed variables that were both not statistically significant and lacked economic significance. The regression results are shown in Table 4.

**Table 4:** Baseline regression results

Variable	Regression coefficients	*t*-value	*p*-value
DID	0.0028	4.1800	0.0000
treat	0.0007	1.3600	0.1730
post	−0.0010	−1.9100	0.0570
lnECO	−0.0029	−9.4800	0.0000
lnENV	0.0028	14.5100	0.0000
energy	−0.0004	−1.6200	0.1070
industry	0.0145	6.1200	0.0000
Constant term	0.0239	7.0200	0.0000
R-squared	0.5530		
Number of obs	480		

The DID coefficient is 0.0028, and it shows a positive correlation with the level of environmental pollution. This indicates that the establishment of the Land Inspection Bureau is associated with a relative increase in local environmental pollution levels. Therefore, during the time period from 2000 to 2015, the first research hypothesis is supported.

Based on the estimation model in Eq. (1), this study reports the DID estimation results of the establishment of the Land Inspection Bureau on local environmental pollution levels. In this process, control variables are introduced to examine the net effect of the establishment of the Land Inspection Bureau, thereby avoiding potential endogeneity issues due to omitted correlated explanatory variables. The results are presented in Table 5.

**Table 5:** Difference-in-differences estimation results

Variable	M1(Y)	M2(Y)	M3(Y)
DID	0.0028 (0.0007)	0.0011 (0.0002)	0.0010 (0.0002)
Control variables	Yes	Yes	Yes
Post	No	Yes	Yes
Year	No	No	Yes
R^2^	0.5530	0.1830	0.2170

*, **, and *** denote significance levels of 10%, 5%, and 1%, respectively, with standard errors in parentheses

In model 1, control variables were introduced while not controlling for fixed effects. The estimated results confirm that the DID estimation coefficient remains significantly positive (*β*_1_ = 0.0028, *p* < 0.01). Furthermore, the study successively controlled for individual effects and time effects. Regression results show that in model 2 (*β*_1_ = 0.0011, *p* < 0.01) and model 3 (*β*_1_ = 0.0010, *p* < 0.01), the estimated coefficients further decrease, but compared to the sign and significance of the coefficients in model 1, there is no fundamental change. This implies that regardless of whether individual and time effects are included, the establishment of the Land Inspection Bureau will exacerbate the level of local environmental pollution, and the estimation results are robust. Based on these robust results, the subsequent research in this paper will fix the time interval of the observation sample from 2000 to 2015.

### Robustness test

1. Parallel trends test

**Fig. 1:**
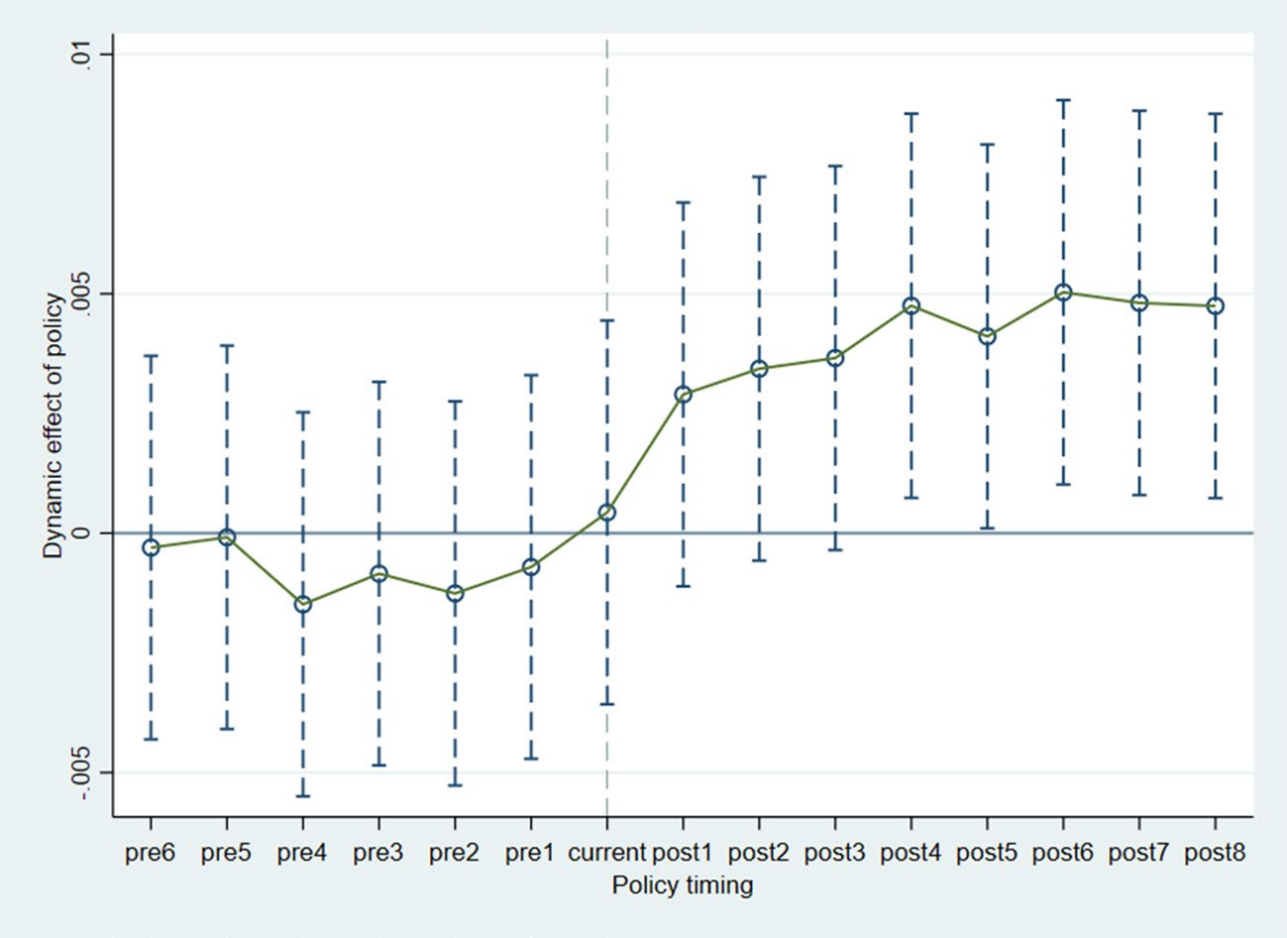
Parallel trends test

The premise for identifying policy effects using the difference-in-differences (DID) method is the fulfillment of the parallel trends assumption. This implies that in the absence of the external policy shock of establishing the Land Inspection Bureau, provinces without the bureau and provinces with the bureau should have similar time trends in environmental pollution levels. Otherwise, it may lead to systematic differences within the sample groups, causing endogeneity issues. To address this, this study regresses interaction terms of dummy variables before and after the establishment of the Land Inspection Bureau as explanatory variables. As is shown in Fig. 1, the regression coefficient for the interaction term before the establishment of the Land Inspection Bureau is not significant, indicating that the pre-treatment group and the control group had similar trends in environmental pollution levels before the establishment. However, the coefficient for the interaction term after the establishment is significantly positive, suggesting that the change in environmental pollution levels for the experimental group before and after the establishment of the Land Inspection Bureau is not purely a time effect but is caused by the establishment of the bureau itself.

(2) PSM-DID model

When analyzing the issue using a difference-in-differences model, it is challenging to maintain a high degree of similarity in the basic characteristics between the experimental group and the control group, which can subsequently affect the scientific rigor of the study. To reduce sampling bias and errors caused by non-random selection, the propensity score matching model is combined with the difference-in-differences method to analyze the environmental protection crowding-out effect of land supervision. The main steps are as follows: Firstly, control variables are selected based on benchmark regression results. Next, the Logit regression model is employed to estimate propensity scores. Subsequently, the nearest neighbor matching method is used to perform one-to-one matching of the experimental group based on these scores. Finally, after completing the matching process, the DID method is applied for analysis.

**Fig. 2:**
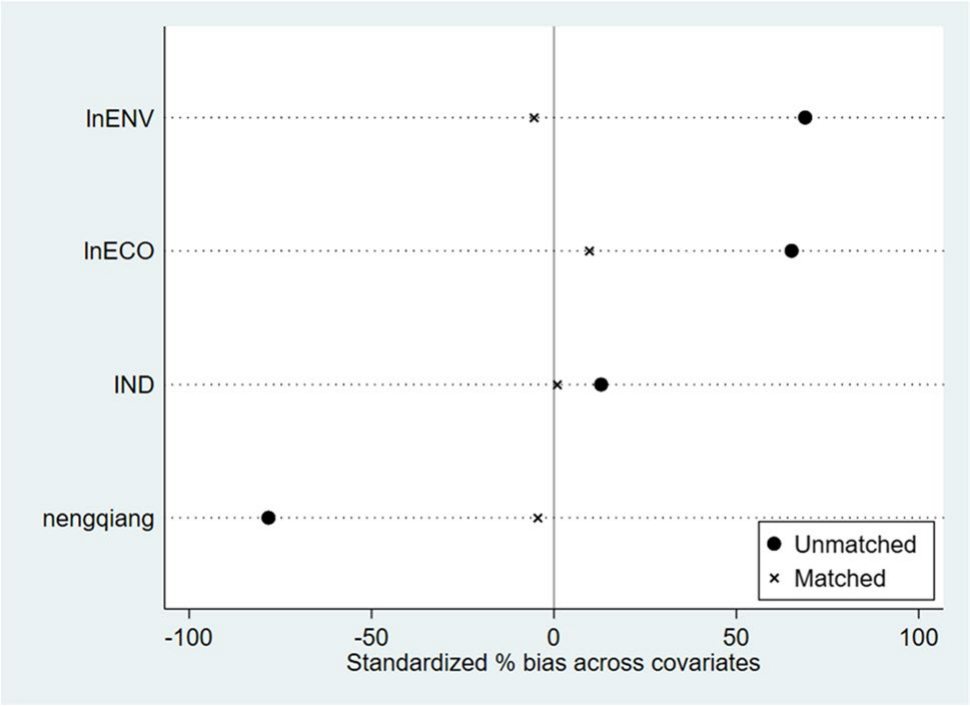
Standard deviation before and after matching for the experimental and control groups (%)

Before conducting the PSM-DID empirical analysis, this study first verifies the matching balance assumption. The matching effectiveness is depicted in Fig. 2, where, compared to before matching, most variables are closer to the vertical line with a standard error of 0. There are no significant differences between covariates, indicating the reasonable suitability of the PSM-DID model used in this study. The results of the PSM matching validity test show that after matching, the standard deviations are all less than 20%, indicating that there are no systematic differences between the experimental group and the control group after matching. The matching method is feasible and meets the matching balance assumption, further confirming the rationality of using PSM-DID for empirical analysis. The PSM-DID regression results show that the coefficient of the core explanatory variable “DID” is 0.0027, significant at the 1% level, indicating that the establishment of the Land Inspection Bureau indeed exacerbates local environmental pollution.

(3) Adding environmental pollution level indicators

In the measurement of environmental pollution levels, different scholars have different preferences. As a test, we first add an indicator for industrial exhaust emissions to the four environmental pollution measurement indicators of the dependent variable in model (1), and re-estimate the model (1). The results are shown in Table 6 below, and it can be observed that the estimated results are still consistent with Table 4, indicating that different measurements of environmental pollution levels do not affect the hypothesis results found in this study.

**Table 6:** Baseline regression results

Variable	Regression coefficients	*t*-value	*p*-value
DID	0.0022	2.7600	0.0060
treat	0.0014	2.2300	0.0260
post	−0.0004	0.7300	0.4670
lnECO	−0.0022	−6.1300	0.0000
lnENV	0.0002	3.5500	0.0000
energy	−0.0011	−3.9500	0.0000
industry	0.0338	15.7000	0.0000
Constant term	0.0239	7.0200	0.0000
R-squared	0.6659		
Number of obs	480		

### Further expansion: heterogeneity testing

Taking into account the actual situation of regional economic disparities in China, especially the fact that the eastern region has attracted a large amount of resources due to its locational advantage, leading to a significantly higher overall level of economic development compared to the central and western regions (Liao et al. 2020), we aim to further examine this regional heterogeneity. We categorize the research sample into eastern and central-western regions based on the geographical location of the province samples, following the latest classification standards by the National Bureau of Statistics of China. Subsequently, we conduct regressions separately for each region, and the results are presented in Table 7.

**Table 7:** Heterogeneity test

Area	DID regression coefficient	*t*-value	*p*-value	*R*-squared	Number of obs
East area	0.0022	2.1800	0.0300	0.7860	176
Midwest	0.0013	1.3600	0.1750	0.4210	304

From the regression results, it is evident that the establishment of the Land Inspection Bureau has a significantly positive impact on eastern provinces. However, for central and western provinces, its impact is statistically insignificant. It can be observed that the influence of the establishment of the Land Inspection Bureau on the level of local environmental pollution exhibits regional heterogeneity. The reasons behind this regional heterogeneity might be twofold. On one hand, there are more provinces in the eastern region with the establishment of Land Inspection Bureaus, and they leverage their robust economic strength and active market environment to drive fiscal revenue for local governments (Zhou et al. 2020), resulting in stronger environmental regulations across various regions. When the establishment of the Land Inspection Bureau affects the intensity of environmental regulations in the host region, the environmental regulation intensity in the non-host region of the eastern region remains strong, making the policy impact more noticeable. On the other hand, the central and western regions have fewer provinces with the establishment of Land Inspection Bureaus, and the overall economic scale in these regions is smaller. The government’s focus and efforts in environmental regulation intensity are not as significant. Consequently, the impact of the Land Inspection Bureau’s establishment on the host region is less pronounced when compared to the non-host region (Sun et al. 2017b). In summary, the research findings validate the third research hypothesis of this study, which suggests that the establishment of the Land Inspection Bureau exhibits regional heterogeneity in its impact on the level of local environmental pollution.

## Analysis of the influencing mechanism

The above verification results indicate that the establishment of the Land Inspection Bureau can influence the level of local environmental pollution. However, what is the specific mechanism through which the establishment of the Land Inspection Bureau affects the degree of environmental pollution? This requires further analysis. As outlined in the research hypothesis above, the establishment of the Land Inspection Bureau primarily affects the intensity of local environmental regulation through its impact on local fiscal revenue. To verify this mechanism, we adopt the mediation testing procedure proposed by Wen et al. (2004) and set up the following testing model:

2 $${\textrm{With}}_{\textrm{it}}={\omega}_0+{\omega}_1{\textrm{DID}}_{\textrm{it}}+{\omega}_2{\textrm{Treat}}_{\textrm{it}}+{\omega}_3{\textrm{Post}}_{it}+{\omega}_4{X}_{it}+{\eta}_i+{v}_t+{\varepsilon}_{\textrm{it}}$$

3 $$\kern0.5em {Y}_{it}={a}_0+{a}_1{\textrm{DID}}_{it}+{a}_2{\textrm{Treat}}_{it}+{a}_3{\textrm{Post}}_{it}+{a}_4{M}_{it}+{a}_5{X}_{it}+{\eta}_i+{v}_t+{\varepsilon}_{it}$$

In Eqs. (2) and (3), *M*_*it*_ represents the mediator variable, while the rest remains consistent with the models mentioned above. Following the testing steps, we conducted regressions on the above equations separately and obtained the regression results shown in Table 8.

**Table 8:** Mechanism testing

Variable	(1)	(2)
	Revenue	Y
DID	−0.0587 *** (0.0049)	0.0043 *** (0.0008)
Revenue		−0.0012*** (0.0046)
Control variable	Yes	Yses
Adj *R*-squared	0.6900	0.5400
Number of obs	480	480

*, **, and *** denote significance levels of 10%, 5%, and 1% respectively

Therefore, the second research hypothesis has been confirmed. According to the regression results, it can be seen that: DID in (1) is significant at the level of 0.01, indicating that the establishment of a land inspectorate will relatively reduce the level of local fiscal revenue; the coefficient of revenue in (2) is significantly negative, indicating that reducing the level of fiscal revenue will increase degree of environmental pollution. Therefore, the second research hypothesis was verified.

## Conclusion

This study utilized panel data from 30 Chinese provinces spanning from 2000 to 2015. It employed the difference-in-differences model to empirically examine the impact of the establishment of Land Inspection Bureaus on the level of local environmental pollution. Additionally, it delved into the mechanisms and heterogeneity of this impact. The study found that the establishment of Land Inspection Bureaus tends to increase the level of environmental pollution in the host regions. This result remained consistent even after undergoing several robustness tests. Therefore, while further strengthening land inspection efforts, it is important to focus on the flexibility of deployment agencies, expand the authority and deterrent power of central dispatch agencies across the country through innovative measures such as rotation and strengthened supervision at the stationed areas, and enhance the effectiveness of inspections.

Mechanism testing results indicated that the establishment of Land Inspection Bureaus can influence the level of local environmental pollution by affecting the local fiscal revenue. Heterogeneity testing revealed that differences in regional economic scales and variations in environmental regulation intensity can influence the impact of Land Inspection Bureau establishment on the environment. In economically larger eastern regions, the impact of Land Inspection Bureau establishment on environmental pollution levels is more pronounced. Heterogeneity analysis shows that the external spillover effect of land inspection on the environment is closely related to regional characteristics. Therefore, the government should design a top-level plan, adapt to local development characteristics, gradually promote the land inspection system in a step-by-step, phased, and hierarchical manner, and overcome specific problems such as short-sightedness in government decision-making.

The exploration in this study provides a new perspective and method for analyzing the environmental regulatory behavior of local governments and land management. Most literature focuses on the interaction between land allocation and fiscal competition among local governments, but mainly examines the effectiveness of land inspection in curbing regional illegal land activities. There is limited research on how local governments adjust the intensity of environmental regulation to cope with fiscal competition pressures brought by central land inspection. Currently, there is less research on policy systems and their implementation effects, mostly focusing on individual policies, and few studies combining environmental protection and land resource policies together, especially paying little attention to the conflicts and contradictions that local governments face in implementing multiple central policies. In addition, there is a lack of research from the micro perspective of local government policy implementation on the macro goal of high-quality economic development. This study considers the “crowding-out effect” of central land inspection on local government environmental protection and incorporates environmental protection and land combination policies into a unified research framework, considering local government behavior decision-making and studying the path to improve policy coordination in the context of high-quality development. This research will make marginal contributions to the theoretical expansion and literature enrichment in fields such as combined policy research on environmental protection and land management, central-local relations, and high-quality development.

Using the establishment of Land Inspection Bureaus as a natural experiment and constructing a difference-in-differences estimation model, this research analyzes the causal relationship between central land inspection and local environmental pollution. These studies provide new research perspectives and methods for analyzing local government’s environmental regulation and land management behaviors. This paper approaches the research from the perspective of local government policy implementation, exploring conflicts between implementing land policies and environmental protection based on the behavioral logic of local governments. This approach can provide new insights and supplements to policy formulation and implementation. By studying the issue of coordination between central policy formulation and local government execution, it will contribute to a more comprehensive understanding of the motives behind local government behavior. It will also help in systematically adjusting the incentive-constraint systems between central and local authorities and the decision-making processes of local governments. This, in turn, will promote high-quality economic development at the macro level through the micro-level actions of local governments.

## Data Availability

The data can be available on request.

## Appendix 1

Calculation of Environmental Pollution Composite Index:

Step 1: Standardize the raw data:

$${P}_{ij}^{\prime \prime }=\frac{X_{ij}-\min \left({X}_{ij}\right)}{\max \left({X}_{ij}\right)-\min \left({X}_{ij}\right)}$$

where *X*_*ij*_ represents the value of the *j*th environmental pollution indicator for the *i*th province (*i* = 1, 2, ..., *m*; *j* = 1, 2, ..., *n*).

Step 2: Perform coordinate shift on the standardized data using the formula: ${P}_{ij}^{\prime }=1+{P}_{ij}^{\prime \prime }$.

Step 3: Calculate the weight *P*_*ij*_ of the *j*th environmental pollution indicator for the *i*th province:

$${P}_{ij}={~}^{{P}_{ij}^{\prime }}\!\left/ \!{~}_{\sum_{i=1}^m{P}_{ij}^{\prime }}\right.$$

Step 4: Calculate the entropy value *e*_*j*_ and coefficient of variation *g*_*j*_ for the jth environmental pollution indicator.

$${e}_j=-\frac{\sum_{i=1}^m{P}_{ij} In{P}_{ij}}{Inm},{g}_j=1-{e}_j$$

Step 5: Calculate the weight of the *j*th environmental pollution indicator in the comprehensive evaluation:

$${W}_j={~}^{{g}_j}\!\left/ \!{~}_{\sum_{j=1}^n{g}_j}\right.$$

Step 6: Calculate the composite evaluation index: ${P}_i=\sum_{j=1}^n{W}_j{P}_{ij}$, where *P*_*i*_ represents the composite evaluation index for the ith province. A higher *P*_*i*_ value indicates a higher level of environmental pollution for that province.

## Appendix 2

Table 9

**Table 9:** Weighting of indicators for 30 provinces from 2000 to 2015

	2000	2001	2002	2003	2004	2005	2006	2007	2008	2009	2010	2011	2012	2013	2014	2015
Industrial wastewater discharge	0.224	0.193	0.201	0.225	0.248	0.260	0.256	0.299	0.295	0.284	0.307	0.313	0.303	0.300	0.303	0.274
Industrial sulfur dioxide emissions	0.223	0.236	0.226	0.237	0.261	0.248	0.240	0.239	0.230	0.233	0.247	0.244	0.240	0.253	0.239	0.217
Industrial solid waste emissions	0.255	0.245	0.265	0.255	0.193	0.207	0.258	0.226	0.225	0.211	0.191	0.223	0.217	0.224	0.232	0.284
Smoke (dust) emissions	0.297	0.326	0.308	0.283	0.298	0.286	0.246	0.236	0.250	0.271	0.255	0.220	0.240	0.223	0.226	0.225
